# Penile calciphylaxis: rare and unrecognized disease

**DOI:** 10.11604/pamj.2022.41.124.33602

**Published:** 2022-02-13

**Authors:** Larbi Hamedoun, Tetou Mohamed

**Affiliations:** 1 Service of Urology, Military Hospital of Instruction Mohamed V, Hay Ryad, Rabat, Morocco

**Keywords:** Penile necrosis, calciphylaxis, end-stage-renal-disease

## Image in medicine

The patient was 78-year-old, a chronic weaned smoker, a poorly balanced type II diabetic, hypertensive and with end-stage chronic renal failure with preserved diuresis, and had been on haemodialysis for more than 10 years. Examination of the external genitalia revealed a blackish, indurated and painful glans penis with the presence of a crust extending to the balanopreputial groove, suggesting necrosis of the penis, which is in favour of dry penile gangrene. This is penile calciphylaxis of pure clinical diagnosis based on careful questioning and examination of the external genitalia. This location is exceptional as the penis is richly vascularised. It is a serious systemic disorder that affects elderly subjects with a history of atherosclerosis, diabetes mellitus, intravascular calcification, 1 to 4% patients with chronic renal disease requiring dialysis, obesity or high blood pressure. Treatment may involve sodium thio-sulfate, wound care, and urinary diversion with a suprapubic catheter to prevent infection, progression of necrosis, and amputation. It is generally of very poor prognosis, with an overall mortality rate of 70%. Patient died the same day following septic shock.

**Figure 1:**
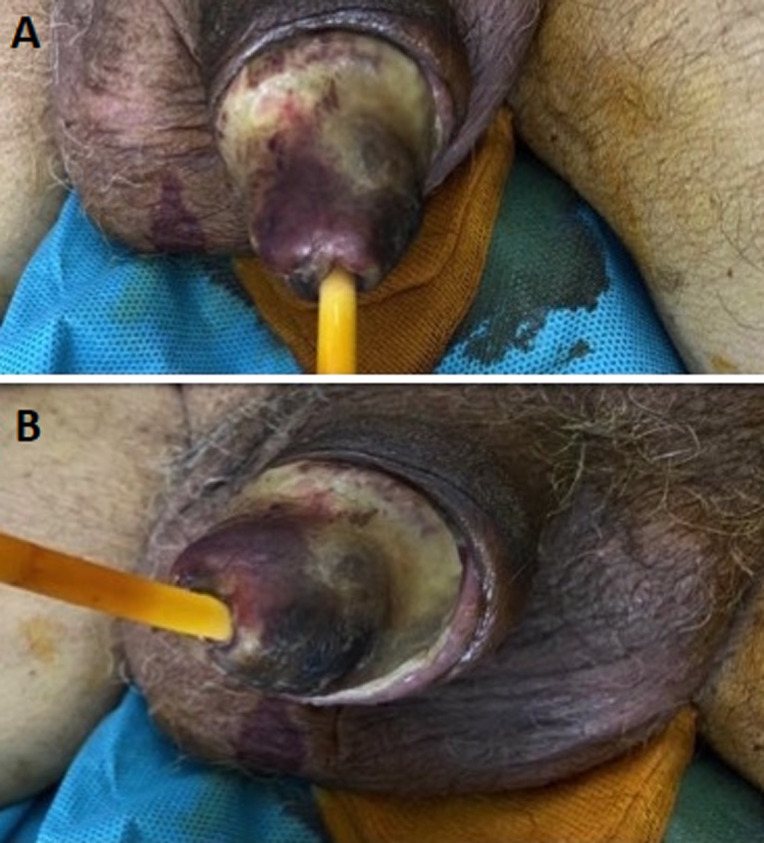
A,B) images during physical exam demonstrating dry penile gangrene

